# Selective COX-2 Inhibitors: A Review of Their Structure-Activity Relationships

**Published:** 2011

**Authors:** Afshin Zarghi, Sara Arfaei

**Affiliations:** *Department of Pharmaceutical Chemistry, School of Pharmacy, ShahidBeheshti University of Medical Sciences, Tehran, Iran.*

**Keywords:** NSAIDs, Cyclooxygenase, Selective COX-2 Inhibitors, Coxibs, SAR

## Abstract

Non-steroidal anti-inflammatory drugs (NSAIDs) are the competitive inhibitors of cyclooxygenase (COX), the enzyme which mediates the bioconversion of arachidonic acid to inflammatory prostaglandins (PGs). Their use is associated with the side effects such as gastrointestinal and renal toxicity. The therapeutic anti-inflammatory action of NSAIDs is produced by the inhibition of COX-2, while the undesired side effects arise from inhibition of COX-1 activity. Thus, it was though that more selective COX-2 inhibitors would have reduced side effects. Based upon a number of selective COX-2 inhibitors (rofecoxib, celecoxib, valdecoxib*etc*.) were developed as safer NSAIDs with improved gastric safety profile. However, the recent market removal of some COXIBs such as rofecoxib due to its adverse cardiovascular side effects clearly encourages the researchers to explore and evaluate alternative templates with COX-2 inhibitory activity. Recognition of new avenues for selective COX-2 inhibitors in cancer chemotherapy and neurological diseases such as Parkinson and Alzheimer’s diseases still continues to attract investigations on the development of COX-2 inhibitors. This review highlights the various structural classes of selective COX-2 inhibitors with special emphasis on their structure-activity relationships.

## Introduction

Non-steroidal anti-inflammatory drugs (NSAIDs) are among the most widely used therapeutics. Through their anti-inflammatory, anti-pyretic and analgesic activities, they represent a choice treatment in various inflammatory diseases such as arthritis, rheumatisms as well as relieving the pains of everyday life. From a historical viewpoint, the first NSAID with therapeutic benefits was aspirin, which has now been used for more than 100 years as a NSAID (1). The cyclooxygenase enzyme was first identified as the therapeutic target of NSAIDs by Vane in 1971, showing that these anti-inflammatory substances block the biosynthesis of prostaglandins (PGs) that contribute to a variety of physiological and pathophysiological functions (2).

*Biochemistry of prostanoids*

**Figure 1:**
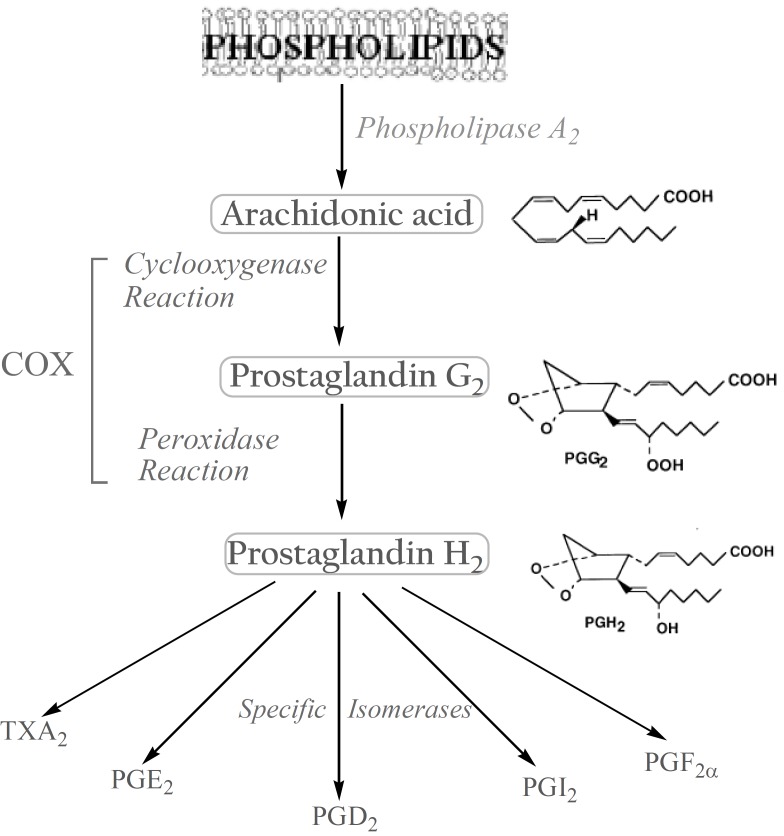
Biosynthesis of prostanoids

The biosynthesis of prostanoids, which include the prostaglandins (PGs) and thromboxanes, occurs in three steps: (a) the mobilization of a fatty acid substrate, typically arachidonic acid (AA), from membrane phospholipids through the action of a phospholipase A2; (b) biotransformation of AA by cyclooxygenase in a bifunctional action which leads to the generation of unstable PGG2 by the cyclooxygenase reaction, and its immediate conversion into PGH2 by the same enzyme in a peroxidase reaction; (c) the conversion of PGH2 to specific prostanoids through the action of synthases and specific isomerases (3). (Figure 1).

*Biological roles of prostaglandins*

Prostaglandins (PGs) are hormone-like bioactive substances mediating autocrine and paracrine signaling over the short distances and are involved in many physiological and pathological processes. They act via high-affinity G protein-coupled receptors: four EP receptors for PGE2 termed EP1-EP4, IP receptor for prostacyclin, DP receptor for PGD2, FP receptor for PGF2*α*. These receptors are linked to the different signal transduction pathways (4). In addition, peroxisome proliferator-activated receptors (PPAR) have been identified as novel intracellular PG receptors (5). Once a prostanoid is formed, it exits the cell and then interacts with G protein-coupled receptors, either on the parent cell or on closely neighboring cells to modulate the second messenger levels (6). Although their tissue distribution depends on the cellular enzymatic material, prostanoids are involved in a very broad range of physiological and pathophysiological responses (7).

In the cardiovascular system, PGD2 and PGE2 as well as PGI2 are potent vasodilators whereas TXA2 displays vasoconstrictor properties. TXA2 also plays a major role in the induction of platelet aggregation while PGI2 presents anticoagulant properties. In the airways, PGF2α and TXA2 are bronchoconstrictors whereas PGI2 and PGE2 act as bronchodilators. In the GI tract, PGE2 and PGF2α as well as PGI2 ensure the protection of the gastric mucosa by lowering the acid secretions, enhancing the mucosal blood flow and stimulating the mucus formation and bicarbonate secretion. TXA2 induces the increased vascular permeability, leading to edema. In the compromised kidney, PGE2 and PGI2, unlike TXA2, stimulate renal blood flow and diuresis. PGE2 and PGF2α, in contrast to PGI2, strongly contract the uterine smooth muscle (8, 9).

Prostanoids also mediate body’s responses to tissue injury or inflammation. PGE2 is the most important PG which mediates the typical symptoms of inflammation: rubor, calor, tumor, dolor and functiolaesa. Dilatation of small blood vessels initiates the development of redness and heat; the increase in vascular permeability causes the characteristic swelling of tissues. It also produces hyperalgesia by a sensitizing action on the peripheral terminals of sensory fibers. Moreover, PGE2 acts on neurons and contributes to the systemic responses to inflammation such as fever, fatigue and pain hypersensitivity (10, 11).

*The COX isozymes*

Despite the wide use of NSAIDs over the last century, their mechanism of action was not fully understood until 1971 when Vane identified their molecular target, the COX enzyme. In the early 1990s, a second isoform (COX-2) was discovered, distinct from the first one, then renamed COX-1. COX-1 and COX-2 are isoenzymes (12). Since isoenzymes are genetically independent proteins, the genes in humans for the two enzymes are located on different chromosomes and show different properties (13). COX-1 is expressed constitutively in many tissues and PGs produced by COX-1 mediate the “housekeeping” functions such as cytoprotection of gastric mucosa, regulation of renal blood flow and platelet aggregation. In contrast, COX-2 is not detected in most normal tissues, but its expression is rapidly induced by stimuli such as proinflammatory cytokines (IL-1b, TNF*α*), lipopolysaccharides, mitogens and oncogenes (phorbol esters), growth factors (fibroblast growth factor, FGF; platelet-derived growth factor, PDGF; epidermal growth factor, EGF), hormones (luteinizing hormone, LH) and disorders of water-electrolyte hemostasis, resulting in increased synthesis of PGs in inflamed and neoplastic tissues. Thus, the inducible isozyme has been implicated in pathological processes such as inflammation and various cancer types (14, 15).

**Figure 2:**
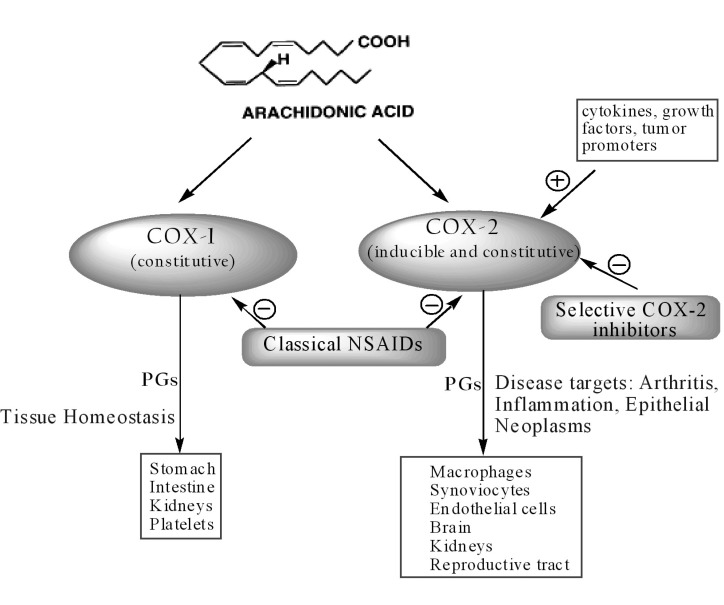
Schematic presentation of the actions of cyclooxygenases (COX-1 and COX-2).

However, recent studies have shown that the relation between the two isoforms is not so straightforward. Indeed, COX-1 may contribute to the inflammation processes whereas COX-2 is constitutively expressed in several tissues and organs such as brain (16), kidneys (17) andreproductive tract (18) (Figure 2).

*Enzymatic structure*

**Figure 3:**
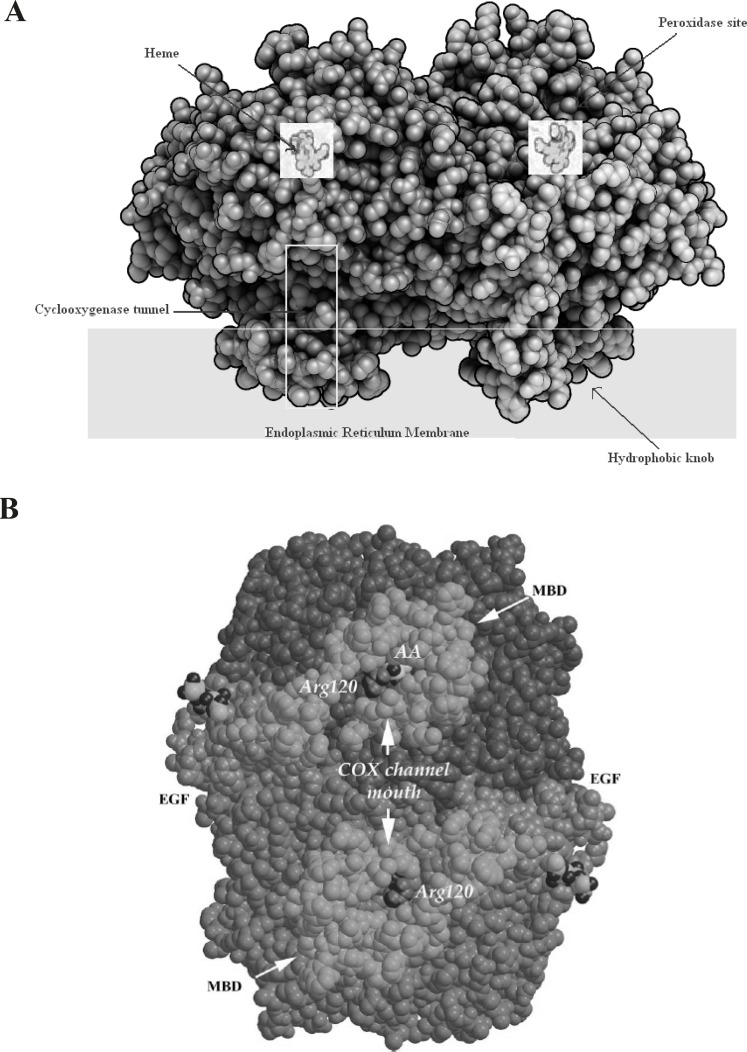
A: Space-filling model of COX-2 along with a Schematic presentation of different parts of cyclooxygenase enzyme. B: A space-filling model of the COX-1 dimer viewed from the membrane plane. The EGF-like and MBD domains are colored green and gold, respectively. The catalytic domains are colored two different shades of blue to highlight the dimer interface. Arg120 (purple), which is part of the channel aperture, defines the beginning of the COX active site. Within one COX channel, a buried AA (yellow and red) is shown (derived from (19).)

The COX isoenzymes are membrane-bound enzymes in the endoplasmic reticulum (ER). The three dimensional structure of the ovine COX-1 was first reported in 1994 and the crystal structures of human and murine COX-2 quickly followed. COX functions as a homodimer and attempts to create monomeric species which have yielded only inactive enzyme. The crystal structures of the COX isoforms are quite structurally homologous and consistent with a high sequence identity (ca. 60%); the overall structures of COX-1 and COX-2 are highly conserved. The COX monomer consists of three structural domains: an N-terminal epidermal growth factor (EGF)-like domain, a membrane binding domain (MBD) of about 48 amino acids in length which anchors the protein to one leaflet of the lipid bilayer, and a large C-terminal globular catalytic domain with the COX active site which accommodates the substrate or the inhibitors and the peroxidase one which contains the heme cofactor. These sites are distinct but functionally and structurally interconnected (19) (Figure 3).

**Figure 4:**
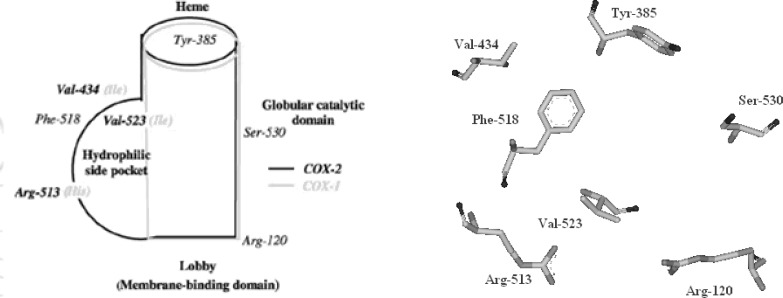
The COX-2 active site and its schematic representation (Figure composed using AccelrysViewerLite 5.0).

The cyclooxygenase active site is created by a long hydrophobic channel that is the site of non-steroidal anti-inflammatory drug binding. This active site extends from the membrane-binding domain (the lobby) to the core of the catalytic domain (20, 21). The arachidonate-binding site is located in the upper half of the channel, from Arg-120 to near Tyr-385. Ser- 530, positioned in the middle of the channel, is the site of acetylation by aspirin (22). Three amino acid differences result in a larger (about 20%) and more accessible channel, in COX-2. The exchange of a valine at position of 523 in COX-2 for a relatively bulky isoleucine (Ile) residue in COX-1 at the same position of the active site of the enzyme, causes a structural modification. This modification in the COX-2 enzyme allows the access to an additional side pocket, which is a pre-requisite for COX-2 drug selectivity. Access to this side pocket is restricted in the case of COX-1. In addition, the exchange of Ile-434 for a valine in COX-2 allows a neighboring residue phenylalanine-518 (Phe-518) to swing out of the way, increasing further access to the side cavity. There is another essential amino acid difference between the two isoforms, which does not alter the shape of the drug-binding site but rather changes its chemical environment. Within the side pocket of COX-2 is an arginine in place of histidine-513 (His-513) in COX-1, which can interact with polar moieties. These differences between the COX active sites have major implications for the selectivity profile of inhibitors (9, 10, 23) (Figure 4).

*The third isoform*

**Figure 5:**
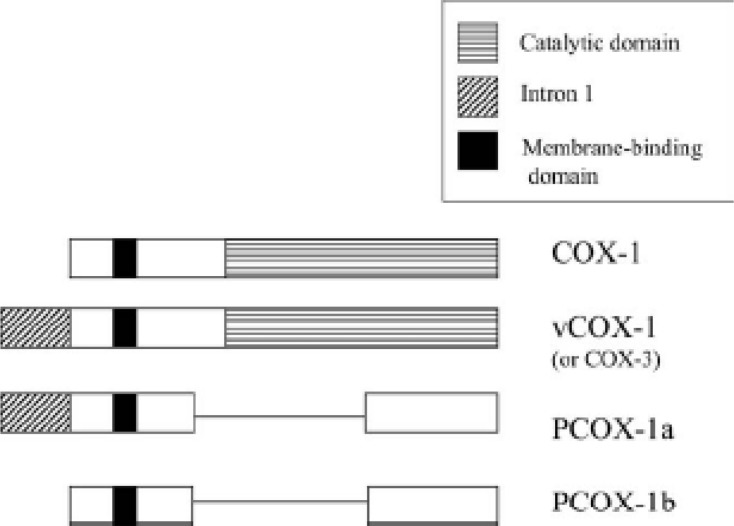
Structure of COX-1 variants produced by alternate splicing (27).

In 2002, the group of Daniel Simmons characterized and cloned a COX enzyme in dog brain which, unlike COX-1 and COX-2, was sensitive to inhibition with paracetamol (acetaminophen). This COX enzyme was a variant of COX-1 and derived from the same gene; it was designated as COX-3 (24). This variant is produced by alternative splicing of the COX-1gene. The only difference is the retention of intron 1 of the COX-1 gene in COX-3 (Figure 5).

COX-3, which contributes about 5% of total COX-1, is a 65-kDa membrane protein whose cyclooxygenase activity is about 80% lower than that of COX-1. This suggests that intron 1 retention may modify the conformation of the active site. Preferential expression of COX-3 in the brain and heart has been reported (24, 25). In addition to COX-3, two shorter variants without cyclooxygenase activity have been identified, PCOX-1a and PCOX-1b (Figure 5). The function of these two inactive, truncated COX-1 variants is unknown (26).

The distinctive characteristic of COX-3 as compared to COX-1 and COX-2 is its greater sensitivity to acetaminophen. Different studies have shown that acetaminophen has only weak inhibitory actions on both COX-1 and COX-2 when tested on *in-vitro *experimental systems. However, it is a potent, selective inhibitor of COX-3 and most likely produces analgesia by inhibiting this enzyme (28). Non-steroidal anti-inflammatory drugs (NSAIDs), such as diclofenacor ibuprofen, are also potent inhibitors of COX-3 expressed in cultured cells, but being highly polar, they are unlikely to reach brain COX-3 in effective concentrations. Moreover, selective COX-3 inhibitors, aminopyrine and antipyrine have been shown to act centrally to cause their antipyretic and analgesic effects in mice.

COX-3 is considered to play a key role in the biosynthesis of prostanoids known to be important mediators in pain and fever. Drugs that preferentially block COX-1 also appear to act on COX-3 (24). However, the existence of COX-3at the nucleotide sequence level in humans has been called to question. A recent sequencing study of the human COX-1 gene found that the first intron contained an additional nucleotide, as compared to canine COX-3. This difference may lead to a frameshift precluding translation into a functional protein (29).

The identification of COX variants opens a new chapter in NSAID pharmacology, which may answer, among other things, how analgesic/antipyretic drugs work. This may lead eventually to the development of new drugs that target thedesired pathway, thereby providing more precise and more effective treatment.

*Side-effects of selective COX-2 inhibitors*

The simplified paradigm of constitutive COX-1 and inducible COX-2 has many exceptions: COX-1 can be regulated during development, whereas COX-2 is constitutively expressed in the brain, reproductive tissues and kidney (30). In addition to its implication in the kidney development, COX-2 plays an important role in the regulation of renal function (perfusion, water handling, and renin release) in both normal and paraphysiological conditions (*i.e*., in patients with liver cirrhosis, renal insufficiency or congestive heart failure). These patients are, therefore, at risk of renal ischemia when NSAIDs and/or selective COX-2 inhibitors reduce vasodilatory PG synthesis (31). Moreover, cyclic hormonal induction of COX-2 is important for ovulation and, at the end of pregnancy, high uterine levels of COX-2 are necessary for the onset of labor. As a result, like for classical NSAIDs, the use of selective COX-2 inhibitors should be avoided in the early stages of pregnancy whereas they should be useful in delaying premature delivery (32, 33).

COX-2 may be involved in the ‘‘adaptativecytoprotection’’ response in GI mucosa. When the latter is inflamed or ulcerated, COX-2 is rapidly induced at sites of injury where it produces large amounts of PGs involved in the healing process. So, selective COX-2 inhibitors should be avoided in patients with gastric susceptibility (34).

In addition, selective inhibitors of COX-2 depress prostacyclin (PGI2), an atheroprotective agent, but not COX-1 derived thromboxane A2 (TXA2), a proaggregatory and vasoconstrictor mediator, which might predispose patients to heart attack and stroke. Thus, the use of these compounds in cardiovascular diseases still requires vigilance (35). Rofecoxib (Vioxx) was withdrawn voluntarily by Merck from the market in September 2004 following the increased cardiovascular risks observed in Adenomatous Polyp Prevention on Vioxx (APPROVe) study. Subsequently, the sale of Bextra (valdecoxib) was also suspended by Pfizer in 2005. This raised a question on the safety of selective COX-2 inhibitors. However, no increased risk of cardiovascular thrombotic events was evident in Celecoxib Long Term Arthritis Safety Study (CLASS) trial conducted on celecoxib (36), which is the only selective COX-2 inhibitor available in U.S. market. A meta-analysis of published and unpublished tabular data from randomized trials revealed that selective COX-2 inhibitors and traditional NSAIDs (high dose regimens of ibuprofen and diclofenac) have similar incidence of adverse cardiovascular events (37). Various studies suggest that the cardiovascular toxicity associated with the use of selective COX-2 inhibitors might be dependent on the dose as well as on the duration of treatment (38). The mechanism underlying the adverse cardiovascular effects associated with the use of COX inhibitors is due to an imbalance between COX-1 derived thrombotic thromboxane A2 (TXA2) in platelets and COX-2 derived vasoprotective prostacyclin (PGI2) in endothelium (36). There should be > 95% suppression of the platelet COX-1 before it can be translated into clinically relevant platelet inhibition (39). All NSAIDs significantly inhibit COX-2 at therapeutic dose but only few traditional NSAIDs (aspirin and naproxen) are able to show > 95% suppression of the platelet COX-1 at such dose. This explains why selective COX-2 inhibitors as well as traditional NSAIDs show adverse cardiovascular effects (40).

It has also been shown that COX inhibition by NSAIDs, besides causing a reduction in the synthesis of vasodilatory and gastroprotective PGs leads to an up-regulation of AA metabolism by the 5-LOX pathway, increasing the formation of LTs and contributing to inflammation and NSAIDs-induced adverse effects such as asthma. Dual inhibition of COX-2 and 5-LOX is, therefore, an interesting alternative to provide safer NSAIDs (9, 41).

*COX-2 and new therapeutic targets*

*COX-2 as a target for anticancer drug development*

Large epidemiological trials studying users and non-users of aspirin have shown that cyclooxygenase (COX) inhibitors and non-steroidal anti-inflammatory drugs (NSAIDs) could be of benefit against the development and growth of malignancies. Moreover, clinical trials in patients with familial adenomatous polyposis syndrome have shown the efficacy of non-selective and selective COX-2 inhibitors in the reduction of the number and the size of colorectal polyps. Celecoxib has been approved by FDA (Food and Drug Administration) as an adjunct for the treatment of familial adenomatous polyposis (FAP) (42).

However, a primary chemopreventive effect has not been demonstrated yet. NSAIDs are also supposed to have a preventive and growth inhibitory effect in extra-colonic epithelial malignancies. Several preclinical studies show promising results with combination treatments of either chemotherapy or radiotherapy with COX inhibitors. Preclinical studies with the simultaneous use of inhibitors of the epidermal growth factor receptor and COX-2 inhibitors have shown also promising results. Encouraging results with the first clinical trials combining chemotherapy with COX-2 inhibitors in patients with cancer in the advanced and neoadjuvant setting have recently been reported. However, NSAIDs effects in cancer cells are mediated not only by COX enzymes but also by interactions with downstream effectors of COX-2 (42).

Hence, we can state that targeting the COX-2 pathway is a promising strategy in the prevention and treatment of solid tumors. Ongoing trials are expected to answer – at least partly – the remaining questions concerning COX-2 and cancer. Here, we focus on the rationale for using selective COX-2 inhibitors as anti-cancer agents.

*Regulating COX-2 expression*

**Figure 6:**
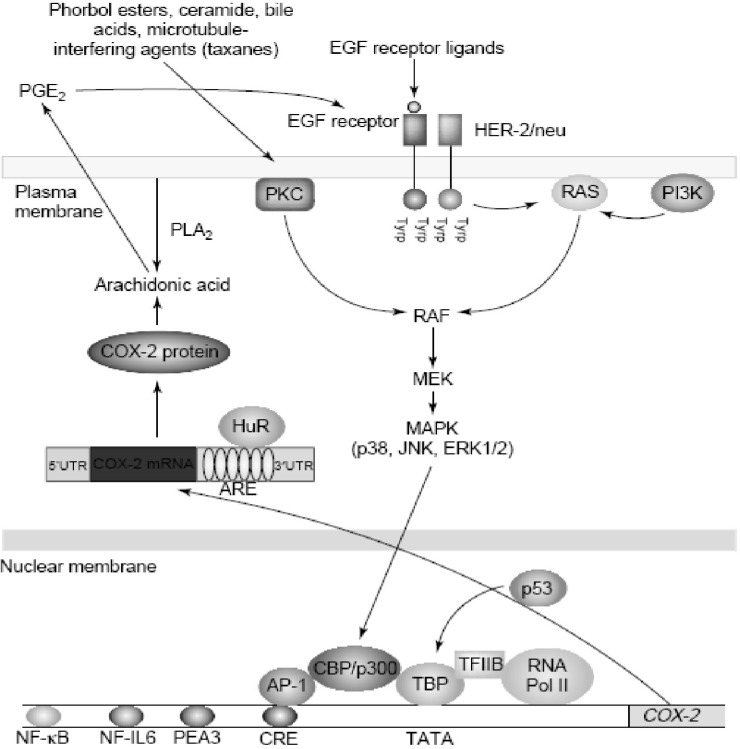
Regulation of cyclooxygenase 2 (COX-2) in cancer.COX-2 is induced by a variety of stimuli including oncogenes (HER-2/neu), growth factors (epidermal growth factor (EGF)), tumor promoters (phorbol esters and bile acids) and chemotherapy (taxanes). Stimulation of either protein kinase C (PKC) or RAS signaling enhances mitogen-activated protein kinase (MAPK) activity, which in turn, activates the transcription of COX-2. Several transcription factors, including activator protein 1 (AP-1) and nuclear factor kB (NF-kB), mediate the induction of COX-2. By contrast, wild-type p53 suppresses transcription of COX-2. COX-2 is also regulated by post-transcriptional mechanisms. The 30-untranslated region (30UTR) of COX-2 mRNA contains a series of sequences (AUUUA) known as AU-enriched elements (AREs) that confer the message instability. Augmented binding of HuR, an RNA-binding protein, to these elements is responsible, at least in part, for increased stability of COX-2 mRNA in tumors. In addition, prostaglandin E2 (PGE2) induces COX-2 by activating the tyrosine kinase activity of the EGF receptor, but it is not known whether this positive feedback mechanism is relevant in human tumors. Abbreviations: CBP, CREB binding protein; CRE, cAMP response element; ERK, extracellular signal regulated kinase; JNK, Jun N-terminal kinase; MEK, MAPK kinase; NF-IL6, nuclear factor interleukin 6; PEA3, polyomavirus enhancer activator 3; PI3K, phosphatidylinositol 3-kinase; PLA2, phopholipase A2; RNA Pol II, RNA polymerase II; TBP, TATA-binding protein (derived from (46)).

Increased amounts of COX-2 are found commonly in both premalignant and malignant tissues (43). Overexpression of COX-2 appears to be a consequence of both increased transcription and enhanced mRNA stability (44, 45). Oncogenes, growth factors, cytokines, chemotherapy and tumor promoters stimulate COX-2 transcription via protein kinase C (PKC) and RAt Sarcoma-mediated (RAS-mediated) signaling (Figure 6). 

Agents that interfere withmicrotubules, including taxanes, induce COX-2 by activating PKC and mitogen-activated protein kinases (MAPKs) (Figure 6). Depending on the stimulus and cell type, a variety of transcription factors including activator protein 1 (AP-1), nuclear factor interleukin-6 (NF-IL6), nuclear factor-kappaB (NF-kB), nuclear factor of activated T-cells (NFAT) and polyomavirus enhancer activator 3 (PEA3) can modulate the transcription of COX-2. Although many factors enhance COX-2 transcription, much less is known about negative modulators. Wild-type, but not mutant, p53 markedly suppresses the transcription of COX-2. These findings suggest that the balance between activation of oncogenes and inactivation of tumor suppressor genes affects expression of COX-2 (46).

There is growing evidence that post-transcriptional mechanisms also determine COX-2 levels in neoplastic tissues. Oncogenes, cytokines, growth factors and tumor promoters induce COX-2 by enhancing mRNA stability in addition to the stimulating transcription. In human colon cancers, overexpression of COX-2 is a consequence of both increased transcription and decreased mRNA turnover. Interestingly, the activation of extracellular signal regulated kinase 1/2 (ERK1/2) and p38 stabilize COX-2 mRNA in addition to the stimulating transcription (46).

*The mechanisms by which COX-2 contributes to cancer*

**Figure 7:**
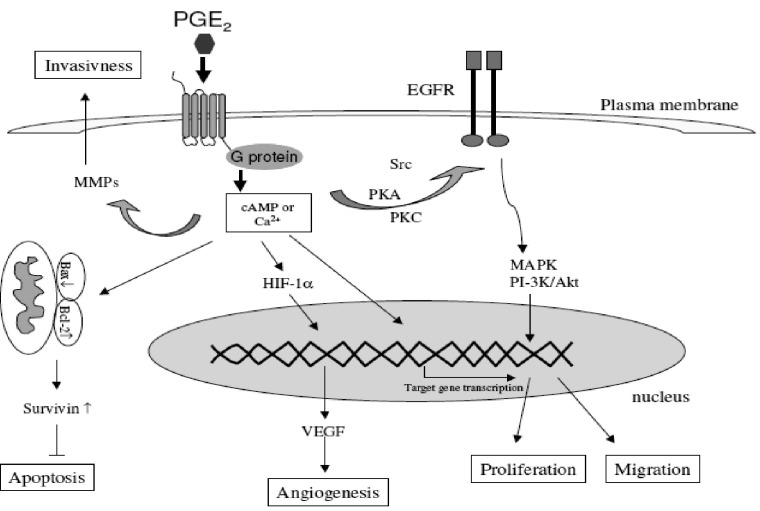
Different mechanisms through which the COX-2-derived prostaglandins are involved in the carcinogenesis (derived from (15)).

COX-2 affects many processes that have been implicated in different stages of carcinogenesis. These include xenobiotic metabolism, cell proliferation, angiogenesis, apoptosis, immune function and tumor invasiveness (15, 42) (Figure 7).

*Activation of carcinogens*

The peroxidase part of COX can convert the procarcinogens to carcinogens and thus initiate tumor formation. Substantial amounts of xenobiotics (natural non-human organic compounds) can be co-oxidized into mutagens by the peroxidase activity of COX (47). This reaction could be relevant at organ sites that are exposed to tobacco carcinogens such as lung, oral cavity and bladder. Similarly, estrogens, oxidized to diethylstilbestrol demonstrate transforming and genotoxic activity. Moreover, the metabolism of the arachidonic acid itself produces mutagens. Some by-products of the oxidation of arachidonic acid, like malondialdehyde are chemically highly reactive and form adducts with DNA (48).

*Prostaglandins and cell proliferation*

Previous studies have demonstrated that PGs stimulate proliferation of different cell lines derived from gastrointestinal tract such as colonic, intestinal, gastric and esophageal cell lines. Therefore, it is not surprising that NSAIDs and selective COX-2 inhibitors as inhibitors of PG synthesis exert the inhibitory effect on the proliferation of malignant cell lines derived from gastrointestinal tract (*in-vitro *studies) and on tumor growth *in-vivo. *Studying downstream mechanisms can also support the role of COX-2 in carcinogenesis (15, 49).

PG E2 concentration is increased in cells with COX-2 overexpression, and is considered as the most important downstream effector of COX-2. Activation of the epidermal growth factor receptor (EGFR) via PG E2 action is of great interest since EGFR is recognized as a therapeutic target in the cancer setting and several EGFR inhibitors have been developed. Their association with COX-2 inhibitors could therefore be interesting in treating cancer (49). EGFR and COX-2 pathways develop a real cross talk: PG E2 stimulates EGFR signaling via the shedding of active EGFR ligands from the plasma membrane but can also induce EGFR transactivation directly via Src pathway stimulation. Conversely, EGFR transactivation stimulates AP-1-mediated induction of COX-2 expression and thus PG E2 expression resulting in a loop of cross-stimulation (42, 50, 51).

*Prostaglandins and apoptosis*

Apoptosis, the morphologically defined form of programmed cell death, plays a crucial role in the carcinogenesis. The disegulation of this process can lead to abnormal survival of cells and the increased risk of mutagenesis and oncogenesis (15).

COX-2-derived PGs regulate programmed cell-death and reduce the apoptotic rate via inhibition of the mitochondrial apoptotic pathway characterized through reduced cytochrome c release, attenuated caspase-9 and -3 activation and upregulation of bcl-2 (15, 52). Additionally, increased prostanoid generation due to the COX-2 overexpression specifically inhibits Fas-mediated apoptosis (53). Another evidence supporting the role of PGs in the regulation of apoptotic rate of tumor cells is the studies demonstrating that COX-2 overexpression in these cells increases their resistance to apoptosis (54). Conversely, COX-inhibitors trigger both the mitochondrial and death receptor-mediated apoptotic pathways with resultant cytochrome c release. In addition, some COX-independent effects on apoptosis have been observed such as inhibition of NFkB signaling via inhibition of IkB kinase B activity and through binding to the nuclear receptors PPAR (55).

*Prostaglandins and increased invasiveness*

Tumor cell invasion is an extremely important factor for the formation of solid tumors and necessary for their spread to distant organs. Matrix degradation and cell motility are essential in this process. Matrix metalloproteinases (MMPs) are a family of matrix degradation enzymes. Their expression is associated with tumor cell invasion of the basement membrane and stroma, bloodvessel penetration and metastasis (42).

It has been demonstrated that COX-2 induces MMP expression in human colon cancer cells and therefore promotes metastasis (56). COX-2-derived PGs play an important role in the increased invasiveness of cancer cells. One of the important mechanisms through which coxibs suppress the tumor invasiveness is the inhibition of matrix metalloproteinases (MMP-2 and MM-9) which are known to facilitate cell invasion and migration with degrading the extracellular matrix (15).

*Prostaglandins and angiogenesis*

Angiogenesis, the formation of new blood vessels from pre-existing vasculature, is an essential process in the carcinogenesis and metastasis. Neovascularization is regulated by the balance between pro-angiogenic factors and angiogenesis inhibitors in the local tissue environment. Important pro-angiogenic factors include vascular endothelial growth factor (VEGF), basic fibroblast growth factor (bFGF), interleukin-8, tumor necrosis factor alpha (TNFα), platelet derived growth factor (PDGF) and COX-derived PGs such as PGE2 and PGI2.

The link between the COX-2-derived PGs and angiogenesis is suggested through studies showing a correlation between COX-2 gene expression and angiogenesis in premalignant tissues and cancer. PGE2 stimulates angiogenesis via the transcription factor hypoxia inducible factor-1 (HIF-1 alpha) leading to the induction of VEGF. On the other hand, VEGF stimulates the COX-2 expression. The ability of COX-2 and VEGF to influence each other suggests a positive feedback amplification mechanism.

While mature blood vessels express COX-1, new angiogenic endothelial cells express the inducible COX-2. Based on these observations, it is hypothesized that tumor-derived growth factors promote angiogenesis by inducing the production of COX-2-derived PGE2 (15, 42).

*Prostaglandins and immune response*

PGs have ability to regulate the immune system. This is of great clinical importance since immunosuppression correlates with the progression of the neoplastic diseases. Macrophages are activated and produce PG E2 which in turn inhibits the production of regulatory cytokines, the B and T-cell proliferation, and decreases the cytotoxic activity of natural killer (NK) cells. Interestingly, the induction of IL-10 and its immunosuppressive effects are related to PG E2 production. Thus, the overproduction of COX-2-derived PGs could result in the inhibition of cell-mediated antitumor response (57).

*Multidrug resistance*

MDR-1 (or P-glycoprotein), is an efflux pump for chemotherapeutic drugs and thereby contributes to multidrug resistance. Overexpression of COX-2 has been found to increase the production and function of MDR-1 in cells in culture, an effect that was prevented by treatment with a selective COX-2 inhibitor. Although much work is required to establish the clinical significance of this interaction, it is appealing to speculate that selective COX-2 inhibitors will enhance the anti-tumor activity of cancer chemotherapy by reducing the multidrug resistance (58).

*Aromatase activity*

Estrogen deprivation is an effective therapy for the prevention and treatment of hormone-dependent breast cancer. The final step in estrogen biosynthesis is catalyzed by aromatase. PGE2 increases the aromatase activity in cells in culture and, thus, should stimulate cell proliferation indirectly by increasing the estrogen biosynthesis. This implies that inhibiting the production of estrogen in breast tissue using a selective COX-2 inhibitor might be useful for either preventing or treating breast cancer (15).

It can be concluded that: 1) COX-2-derived PGs play a key role in the tumorogenesis; 2) The tumor-promoting effect of PGs may be attributed to their ability to stimulate the cell proliferation and migration, to inhibit the apoptosis and to increase angiogenesis and invasiveness; 3) in accordance to the proposed major role of COX-2 in cancerogenesis, selective COX-2 inhibitors have been shown in numerous studies to exhibit strong chemopreventive effect on the development of cancers.

*COX-2 and neurological diseases*

COX-2 in CNS may have an ambivalent functionality since the basal production of PGs through COX-2 may participate in neuronal homeostasis, whereas the expression of COX-2 is associated with brain development.

*Alzheimer’s disease*

Alzheimer’s disease (AD) is among the most important health care problems worldwide. The neuropathological features of Alzheimer’s disease (AD) include the accumulation of microglia around plaques, a local cytokine-mediated acute-phase response, and activation of the complement cascade. This inflammatory response may damage neurons and exacerbate the pathological processes underlying the disease. A large number of epidemiological studies have indicated that the use of NSAIDs may prevent or delay the clinical features of AD. Since COX-2 expression in the brain and PGE2 content in the cerebrospinal fluid have been reported to be elevated in AD together with the finding that COX-2 protein levels in the brain correlate with the severity of amyloidosis and clinical dementia, it has been suggested that COX-2 inhibition by NSAIDs might be involved in the apparent protection in this setting. However, the results of a recent randomized, double-blind clinical trial of rofecoxib vs. naproxen have failed to demonstrate a significant slowing of cognitive decline in patients with mild-to-moderate Alzheimer’s disease over 12 months. Several factors might have contributed to the failure of this trial. In particular, the selection of patients with advanced neuropathology and the short period of exposure to treatment may have played a role. Alternatively, COX-independent mechanisms of NSAIDs may have contributed to the apparent protection demonstrated in epidemiological studies. It has been reported that NSAIDs may activate the peroxisome proliferator-activated receptor (PPAR). In fact, PPARγ activation leads to the inhibition of microglial expression of a broad range of pro-inflammatory molecules. However, it should be pointed out that no evidence is available to correlate these alternative mechanisms of NSAIDs with their clinical benefit reported inpopulation-based studies.

COX-2 is constitutively expressed at high levels in brain and is specifically concentrated in pyramidal neurons which are vulnerable to AD pathology. On the other hand, COX-1 is not constitutively expressed in brain at high levels but is upregulated in reactive microglia, the target for inflammatory suppression. So far, COX-2 has not been detected in astrocytes and microglia in AD and is barely induced with the inflammatory mediators in AD. It would be anticipated, therefore, that NSAIDs 1 rather than selective COX-2 inhibitors would be more likely to reduce the brain inflammation selectively (41, 59, 60).

*Parkinson disease*

It has been shown that NSAIDs reduce the dopaminergic neuron degeneration in animal models of Parkinson disease (PD), but no epidemiological data are available on NSAID use and the risk of PD. However, it has been shown that COX-2 is up-regulated in brain dopaminergic neurons of both PD postmortem specimens and 1-methyl-4-phenyl-1,2,3,6-tertrahydropyridine (MPTP) mouse model of PD, and COX-2 inhibition prevents the formation of the oxidant species dopamine-quinone involved in the pathogenesis of PD, suggesting that the inhibition of COX-2 may be a valuable target for the development of new therapies for PD aimed at slowing the progression of the neurodegenerative process (61).

*COX-2 inhibitors*

**Figure 8:**
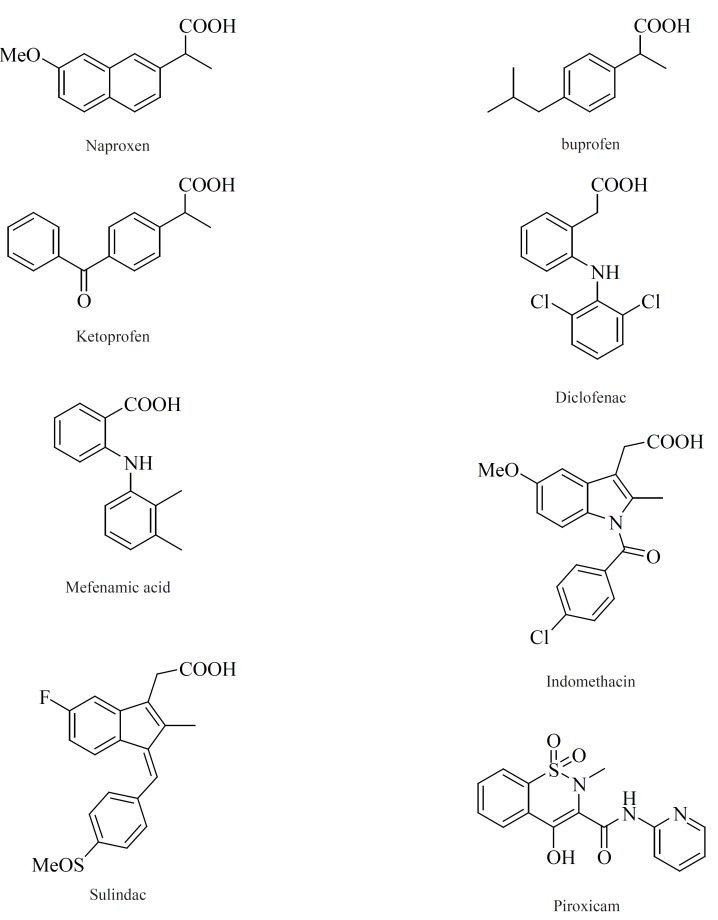
NSAIDs

Within the last two decades, the volume of literature on the structural types introduced as selective COX-2 inhibitors is enormous. In this review, we have chosen to focus on the structure-activity relationship (SAR) and also various structural families of compounds, which have emerged within the last years. The chemical structures of COX-2 inhibitors are heterogenic so that a further classification of this group will be made in the following chapter. Contrary to the classic NSAIDs (Figure 8), this new class of enzyme inhibitors is lacking a carboxylic group, thus effecting COX-2 affinity by a different orientation within the enzyme without formation of a salt bridge in the hydrophobic channel of the enzyme.

In general classification, selective COX-2 inhibitors belong to two major structural classes: 1) Tricyclics (also known as ortho-diarylheterocycles or carbocycles); 2) Non-tricyclics.

*Tricyclics*

**Figure 9:**
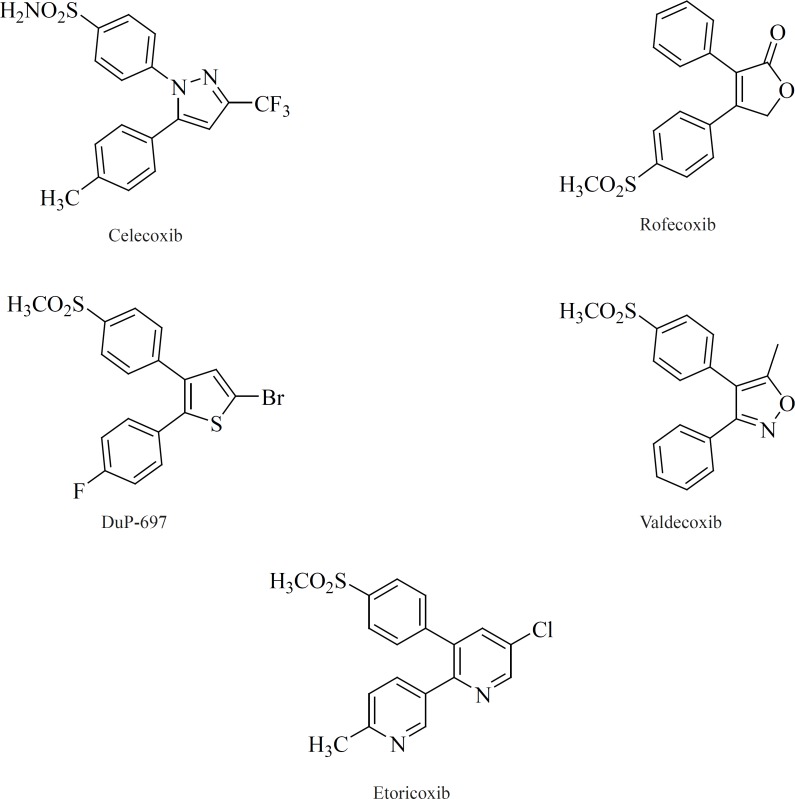
Coxibs

All of the compounds in this class possess 1,2-diarylsubstitution on a central hetero or carbocyclic ring system with a characteristic methanesulfonyl, sulfonamido, azido, methanesulfonamide or pharmacophore-based tetrazole group on one of the aryl rings that plays a crucial role on COX-2 selectivity. Coxibs such as Celecoxib, Rofecoxib, Valdecoxib and *etc*, belong to this common structural class (62) (Figure 9).

Compounds belonging to this class can be sub-classified based on the size and type of the central heterocyclic or carbocyclic ring system (core). 4-, 5- and 6-membered rings and also bicyclic and tricyclic fused and spiro ring systems have frequently been used as the central core for this group of compounds.

*a) 4-membered cores*

**Figure 10:**
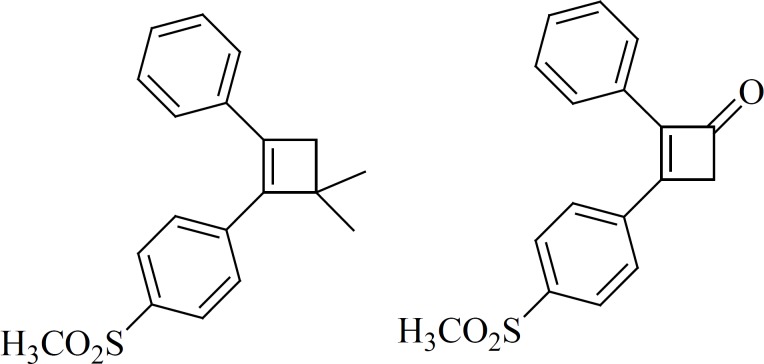


Ring contraction to smaller carbocycles such as cyclobutenes also leads to potent COX-2 inhibitors as well as insertion of 5- and 6-membered carbocyclic or heterocyclic groups (Figure 10). Compounds with a cyclobutene central ring show IC50-values for COX-1 of 0.12 [1] and > 5 mmol [2], for COX-2, 0.002 [1] and 0.11[2] μM (63).

*b) 5-membered cores*

A wide variety of 5-membered heterocycles can serve as a template for COX-2 inhibitors (Figure 13), *i.e. *pyrrole [3] (64), pyrazole (celecoxib, [4], [5]) (65), (62), thiazole [6] (66), oxazole [7], [8] (67), oxadiazole [9] (68), furanone (rofecoxib, [10]) (69, 70), imidazole [11], [12] (71,72), isoxazole (valdecoxib), triazole [13] (73) and thiophene (DuP 697).

Knaus*et al*. reported a series of methanesulfonamide analogues of rofecoxib which in general, show decreased COX-2 inhibitory potency and selectivity in comparison with rofecoxib. Compound [10] was the most promising analogue among the synthesized analogues (COX-2, IC50 = 0.9 μM; SI > 111).

**Figure 11:**
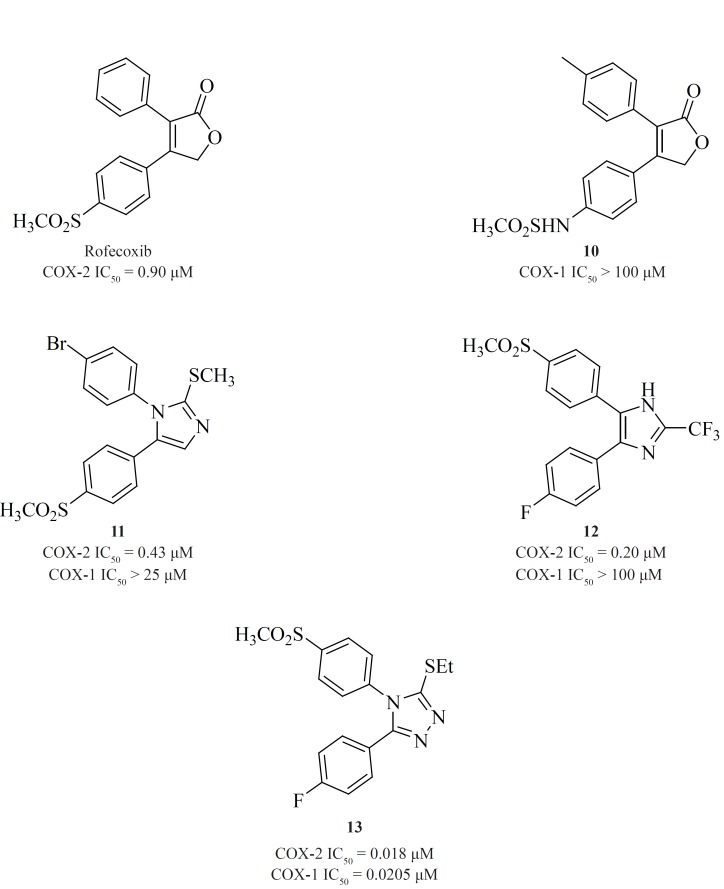


Zarghi *et al*. reported a novel series of 2,3-diaryl-1,3-thiazolidine-4-ones possessing a SO2Me pharmacophore at the para-position of C-2 phenyl ring in conjunction with a substituent (H, F, Cl, Me and OMe) at the para-position of the N-3 phenyl ring. Compounds [14] and [15] were potent COX-2 inhibitors which showed higher selectivity than celecoxib (74). Besides, a group of 2-aryl, 3-benzyl-(1,3-oxazolidine or 1,3-thiazolidine)-4-ones possessing a SO2Me pharmacophore at the para-position of C-2 phenyl ring were reported by Zarghi*et al*. Compound [16] (COX-2, IC50 = 0.21 μM; SI > 476), has a higher selectivity index than celecoxib. Compound [17] has lower potency and selectivity (COX-2, IC50 = 0.32 μM; SI > 312.5), which suggests that COX-1/-2 inhibition in this scaffold is sensitive to the type of heteroatom (O, S) (75) (Figure11).

New series of 2,4,5-triarylimidazoles possessing a SO_2_CH_3 _pharmacophore at thepara position of C-4 phenyl ring has also been reported by Zarghi*et al*.

**Figure 12:**
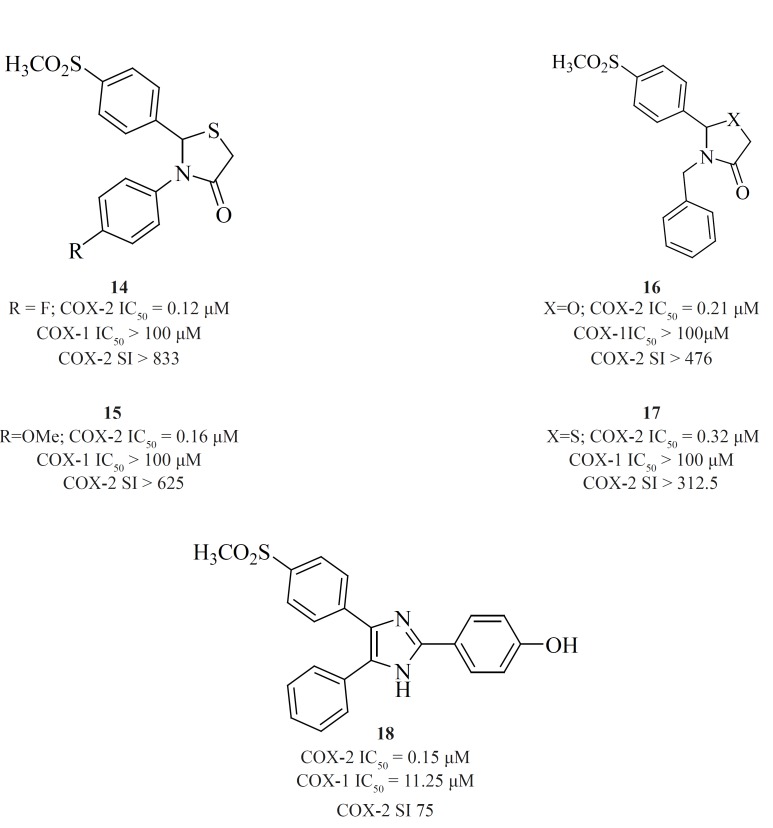


Structure-activity relationship of this group showed that COX-2 inhibitory potency and selectivity is dependent on the nature of the substituent on the C-2 phenyl ring. The order of selectivity was OH > F >OMe>H , Me >NHCOMe> Cl. Compound [18] possessing OH group at the para-position of the C-2 phenyl ring is the most potent and selective COX-2 inhibitor in this group (76) (Figure 12).

*c) 6-membered cores*

**Figure 13:**
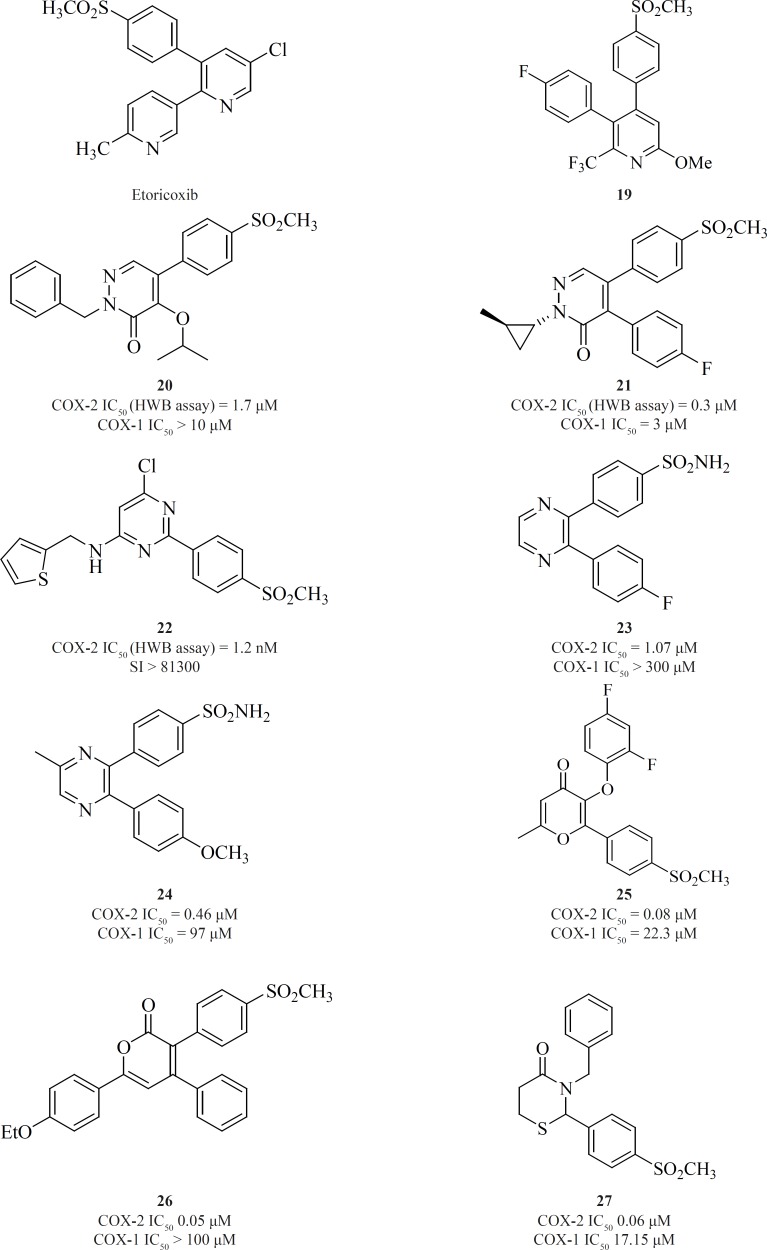


One of the first structural types emerged in this category were pyridine series. 1,2-diarylpyridine derivatives (such as etoricoxib) and 2,3-diarylpyridine derivatives [19] have shown good COX-2 inhibitory potencies and selectivities (77) (Figure 13).

Li *et al. *described the SAR studies on a new class of orally active COX-2 inhibitors based on the six-member heterocyclic pyridazinone system. Various *n*-substituted analogues were initially prepared to evaluate the effect of *n*-substitution in this category. It was very clear that *n*-substitution was absolutely required for a good *in-vitro *COX-2 inhibitory potency since the unsubstituted analogues were not potent. An increase in the size of nitrogen substituent improved COX-2 inhibitory potency. Two potent and selective COX-2 inhibitors [20] and [21] have been identified from the pyridazinone template. These two compounds also showed excellent efficacy in animal models of anti-inflammation, the rat paw edema and rat pyresis assays (78) (Figure 13).

Pyrimidine-based COX-2 inhibitors were introduced by Orjales*et al. *Compound [22] was also the most selective pyrimidine derivative of the series with a calculated HWB selectivity index of 81,300 (780-fold higher than rofecoxib) (79) (Figure 13).

Singh *et al. *reported 2,3-diarylpyrazines having 4-methylsulfonyl/sulfonamide phenyl pharmacophore. Compounds [23] and [24] were the most selective COX-2 inhibitors in this group (80). The 2,3-diarylpyrane-4-ones [25] and 3,4-diarylpyrane-2-ones [26] containing a para-methylsulfonylpharmacophore have also shown to be the proper scaffolds for selective COX-2 inhibitors with potent oral anti-inflammatory activity (81, 82) (Figure 13).

Zarghi *et al. *reported a group of 2,3-diaryl-1,3-thiazolidine-4-ones possessing a COX-2 SO2Me pharmacophore at the para-position of C-2 phenyl ring in conjunction with different substituents at the para-position of the N-3 phenyl ring. Compound [27] was the most potent and selective COX-2 inhibitor among this group of compounds. It was as potent as celecoxib (COX-2 IC_50 _= 0.06 μM; S.I. = 405), in terms of COX-2 inhibitory activity but showed less selectivity (83) (Figure 13).

*d) Fused bicyclic cores*

**Figure 14:**
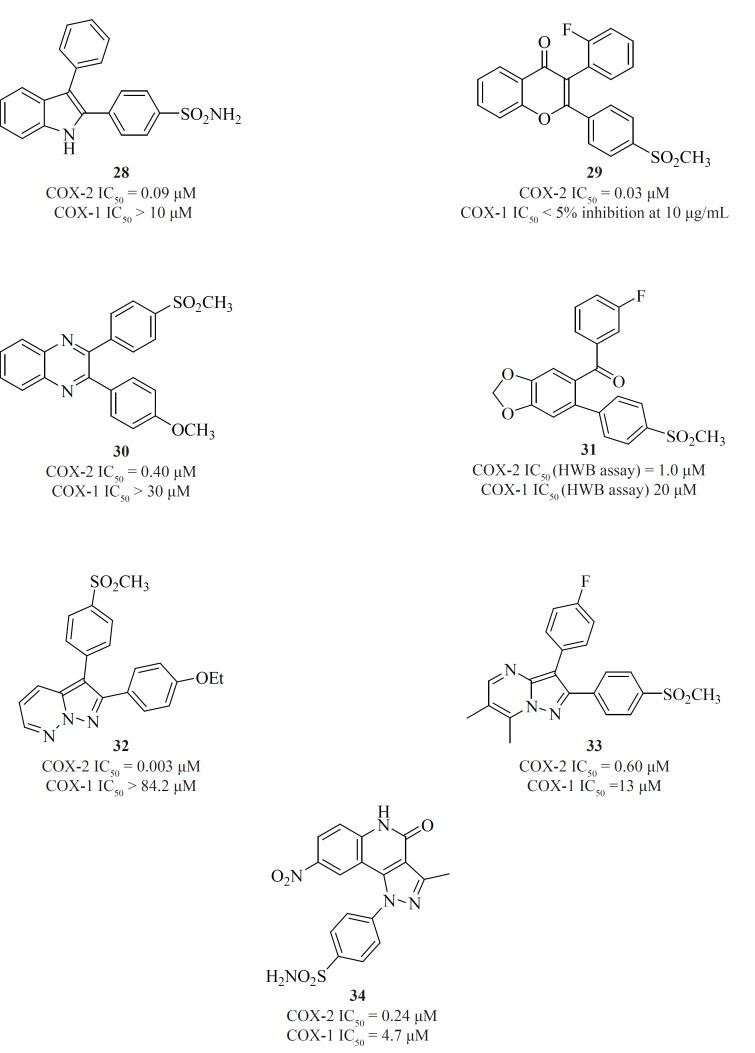


Apart from single 4-, 5- or 6-membered heterocyclic or carbocyclic rings, numerous examples of bicyclic or fused-ring systems as the central template have also appeared in the literature. Indoles [28] (84), 2,3-diaryl-4H-chromen-4-ones [29] (85), 2,3-diarylquinoxalines [30] (81), benzo-1,3-dioxolanes [31] (86), pyrazolo[1,5-b]pyridazines [32] (87), pyrazolo[1,5-a]pyrimidines [33] (88) and pyrazolo[4,3-c]quinolinones [34] (89) are a few examples (Figure 14).

**Figure 15:**
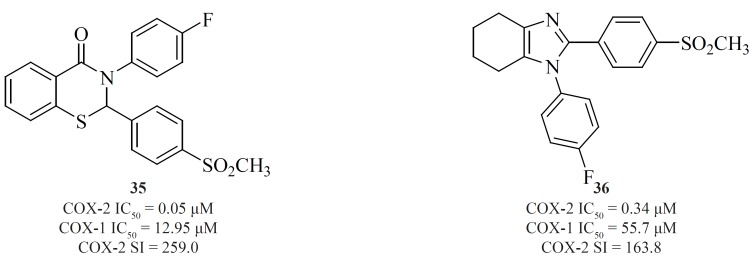


Zarghi *et al. *reported new 1,3-benzthiazinan-4-one derivatives possessing a sulfonylmethylpharmacophore at the para-position of C-2 phenyl ring. In this class of compounds, COX-1/-2 inhibition is sensitive to the nature of the N-3 substituents, and 3-(4-fluoropheny)-2-(4-methylsulfonylphenyl)-1,3-benzthiazinan-4-one [35] exhibited high COX-2 inhibitory potency and selectivity (90) (Figure 15).

A new group of 4,5,6,7-tetrahydro-1H-benzo[d]imidazoles possessing a SO_2_CH_3_ pharmacophore at the para-position of C-2 phenyl ring has been reported by Zarghi*et al*. Compound [36] possessing a fluoro atom at the para-position of N-1 phenyl ring is the most potent and selective COX-2 inhibitor in this group (91) (Figure 15).

Zarghi*et al. *also introduced quinoline as the central core template for potent and selective COX-2 inhibitors. In a group of 4-carboxyl quinoline derivatives possessing a methylsulfonyl COX-2 pharmacophore at the para-position of the C-2 phenyl ring in conjunction with various substituents at C-7 and C-8 quinoline ring, compound [37] was the most potent and selective COX-2 inhibitor, with potency and selectivity higher than the reference drug, celecoxib (92).

**Figure 16:**
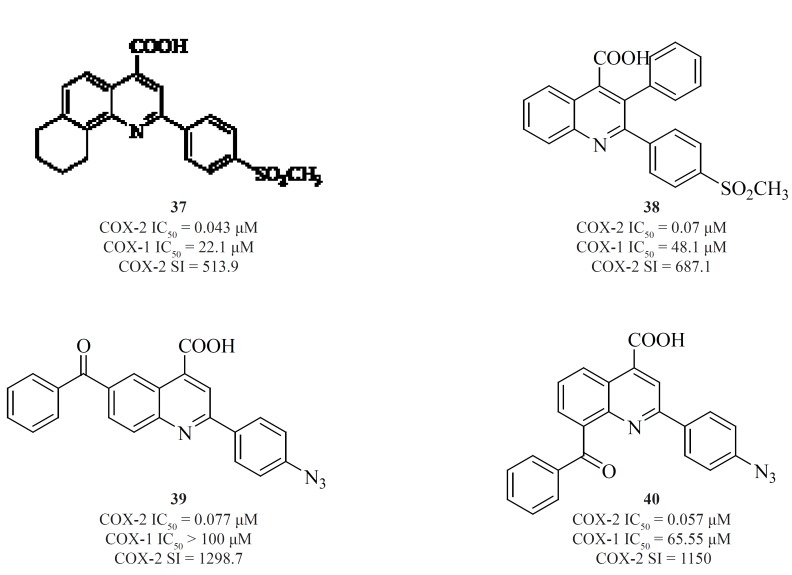


Furthermore, in a series of 2,3-diarylquinoline derivatives, compound [38] possessing a carboxyl group at C-4 position of the quinoline ring, showed the highest COX- 2 inhibitory potency and selectivity (93). Zarghi*et al. *also reported 2-(4-(substituted) phenyl)quinoline-4-carboxylic acid derivatives possessing benzoyl moiety at C-6 or C-8 position of the quinoline ring. The rational for the design of these compounds was based on ketoprofen structure as a part of 2-aryl-quinoline-4-carboxylic acid scaffold. Compound [39] was the most selective COX-2 inhibitor in this group (COX-2 IC_50 _= 0.077 μM; S.I. = 1298), with selectivity index of higher than the reference drug, celecoxib (COX-2 IC_50_ = 0.06 μM; S.I. = 405). Its regioisomer, compound [40] is also a very potent and selective COX-2 inhibitor in this series of compounds (94) (Figure 16).

*Non-tricyclics*

In addition to the classical tricyclic COX-2 inhibitors such as Coxib family, there are several non-classical structures which we here classify as non-tricyclics. These series of compounds lack the cyclic central core. Instead, they possess acyclic central systems such as olefinic, iminic, azo, acetylenic and *α,β*-unsaturated ketone structures. The central acyclic core may contain a two-membered or three-membered chain structure which is the basic point for sub-classification of these compounds.

a) *Non-tricyclics with a two-membered central template*

**Figure 17:**
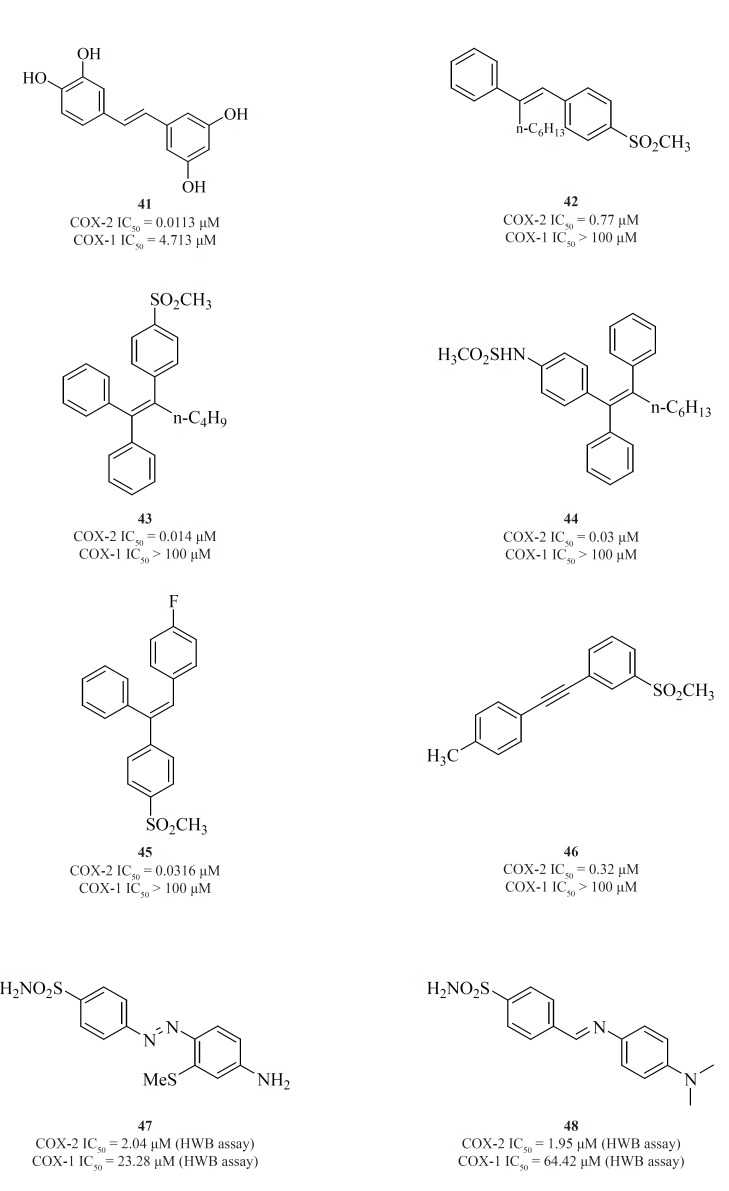


The 1,2-diarylethenes such as natural resveratrol analogues [41] (95) and also 2-alkyl-1,2-diarylolefines [42] (96), 1,1,2-triarylethenes [43], [44], [45] (97-99), acetylenes [46] (100), phenylazobenzenesulfonamides [47] (101) and 4-phenyliminoethyl benzenesulfonamides [48] (102) are included in this group (Figure 17).

Knaus*et al*. introduced series of triaryl (*Z*)-olefins, in which COX-2 inhibitory potency and selectivity increased considerably with the increase in 2-alkyl chain length (up to 4 carbons). *N-Butyl *substituted compound [43] exhibited excellent potency and selectivity better than celecoxib. In addition, in series of 2-alkyl-1,1,2-triaryl (*Z*)-olefins possessing *para*-MeSO2NH/N3 as COX-2 pharmacophoric feature on the C-1 phenyl ring, compound [44] was the best, exhibiting potency comparable to celecoxib. In series of 1,1,2-triaryl (*E*)-ethenes having para-methylsulfonyl moiety on the C-1 phenyl ring, substitution at the C-2 phenyl ring with 4-fluoro substituent afforded [45] with better inhibitory potency and selectivity than celecoxib (Figure 17).

Knaus*et al*. reported a group of 1-((methylsulfonyl)phenyl)-2-phenylacetylene regioisomers in which the COX-2 SO_2_Me pharmacophore was located at the para-, meta- or ortho-position of the C-1 phenyl ring on a linear acetylene template. The SAR data show that the isozyme selectivity in these compounds is dependent on the position of SO_2_Me group on C-1 phenyl ring, as well as the nature of the substituent on C-2 phenyl ring. Compound [46] was the most potent and selective COX-2 inhibitor among these compounds.

b) *Non-tricyclics with a three-membered central template*

**Figure 18:**
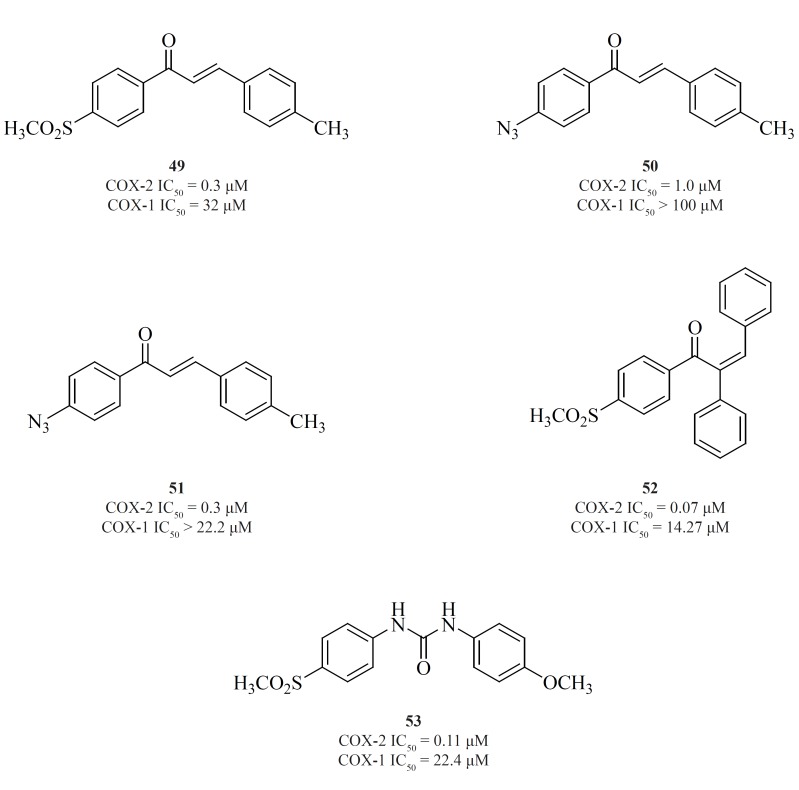


Chalcone derivatives are one of the most important groups of compounds in this category. Zarghi*et al*. for the first time reported a group of (E)-1,3-diarylprop-2-en-1-one regioisomers possessing a COX-2 SO_2_Me pharmacophore at the para-position of C-1 or C-3 phenyl ring in conjunction with a substituent (H, Me, F, and OMe) at the para-position of the other phenyl ring. SAR studies showed that the presence of SO_2_Me on C-1 phenyl ring results in better COX-2 inhibitory potency and selectivity. Compound [49] was the most potent and selective COX-2 inhibitor in this group (103). Compounds possessing N_3_ and NHSO_2_Me pharmacophores, [50] and [51], were introduced afterwards (104) (Figure 18).

 In the continuation of this research, Zarghi*et al. *also described the synthesis and biological evaluation of a group of acyclic (E) and (Z)-1,2,3-triaryl-2-propen-1-ones possessing a methylsulfonyl COX-2 pharmacophore at the para-position of the C-1 phenyl ring in conjunction with various aryl substituents at C-3 propenone moiety. In these designed compounds, we utilized prop-2-en-1-one scaffold instead of olefin moiety in 1,1,2-triaryl olefins. The SAR studies indicated that in this class of compounds, COX-1/-2 inhibition is sensitive to the geometry of propenone and the type of substituent at the C-3 of the propenone moiety. The Z isomers appeared to be more potent and selective inhibitors of the COX-2 isozyme. (Z)-1-(4-(methylsulfonyl) phenyl)-2,3-diphenylprop-2-en-1-one [52] showed the most potency and selectivity on COX-2 inhibition (105) (Figure 18).

A group of 1,3-diarylurea derivatives possessing a methylsulfonylpharmacophore at the para-position of the N-1 phenyl ring having a variety of substituents (H, F, Cl, Me, OMe) at the para-position of the N-3 phenyl ring were also reported by Zarghi*et al*. The SAR results showed that the presence of a hydrogen acceptor group such as methoxy or fluorine substituent at the para-position of the N-3 phenyl ring may improve the selectivity and potency of COX-2 inhibition. Compound [53] was the most potent and selective COX-2 inhibitor in this group (106) (Figure 18).

*Modified NSAIDs*

**Figure 19:**
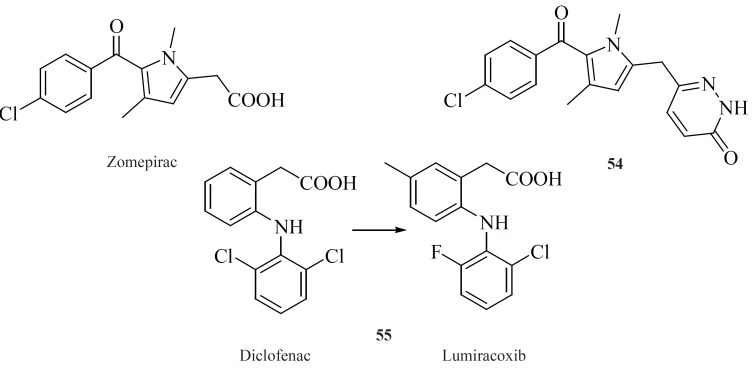


Modifying well known NSAIDs into selective COX-2 inhibitors represents an interesting strategy. Indomethacin, zomeoirac [54] (107), diclofenac and many other NSAIDs have been successfully elaborated into the selective COX-2 inhibitors. Novartis group described conversion of diclofenac into lumiracoxib [55], which exhibits 500-fold selectivity for COX-2 over COX-1 (108) (Figure 19).

Amongst the NSAIDs studied so far, the indomethacin template appears the most flexible in delivering COX-2-specific inhibitors following the functional group manipulations. However, the methodology utilized in NSAID modification does not follow a general scheme. Various attempts have been made to shift the enzyme selectivity of indomethacin from COX-1 to COX-2 while keeping the potency on the same level and reducing the unwanted side-effects at the same time. In principle, the strategy consisted of introducing larger substituents to fit into the active site volume of COX-2 (10).

**Figure 20:**
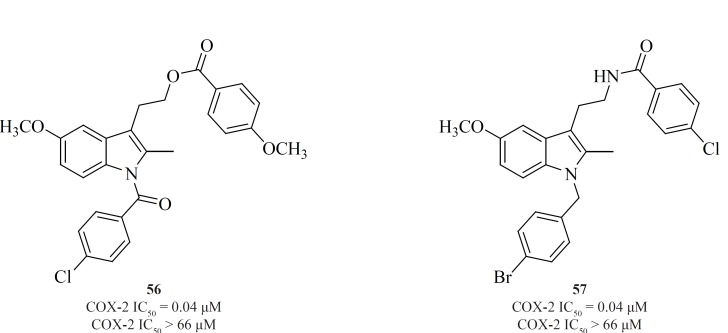


Conversion of non-selective NSAIDs to esters and amides is a facile strategy for generating COX-2 inhibitors from known drugs but it has the limitation that indomethacin esters and possibly some amides may be hydrolyzed to indomethacin *in-vivo*. Therefore, indolyl esters and amides with essentially the ‘‘reverse’’ orientation have been reported that selectively inhibit COX-2. Such compounds eliminate or minimize the generation of indomethacin *in-vivo*. Compounds [56] and [57] are the most potent and selective COX-2 inhibitors resulted from this strategy (109) (Figure 20).

## Conclusions

The development of selective COX-2 inhibitors started in early 1990’s with the identification of COX-2 isoenzyme which was found to be responsible for the pathological processes such as inflammation and pain. Thus, it was though that more selective COX-2 inhibitors would have reduced the side effects. Moreover, recent studies indicating the place of COX-2 inhibitors in cancer chemotherapy and neurological diseases such as Parkinson and Alzheimer’s diseases still continues to attract investigations on the development of COX-2 inhibitors. In this review, the main emphasis was on the structure-activity relationship (SAR) and also various structural families of compounds, which have emerged within the last decade. In general classification, selective COX-2 inhibitors belong to two major structural classes: 1) Tricyclics, 2) Non-tricyclics. All of the tricyclic compounds possess 1,2-diarylsubstitution on a central hetero or carbocyclic ring system with a characteristic methanesulfonyl, sulfonamido, azido, methanesulfonamide or tetrazolepharmacophore group on one of the aryl rings that plays a key role on COX-2 selectivity. Non-tricyclics lack the cyclic central core. Instead, they possess acyclic central systems such as olefinic, iminic, azo, acetylenic and *α,β*- unsaturated ketone structures. The central acyclic core may contain a two-membered or three-membered chain structure.
